# Supplementary figures and images for: Toxoplasma gondii phosphatidylserine flippase complex ATP2B-CDC50.4 critically participates in microneme exocytosis
Source: PLoS Pathog. 2022 Mar 24;18(3):e1010438. doi: 10.1371/journal.ppat.1010438 (PMC8982854; doi:10.1371/journal.ppat.1010438)

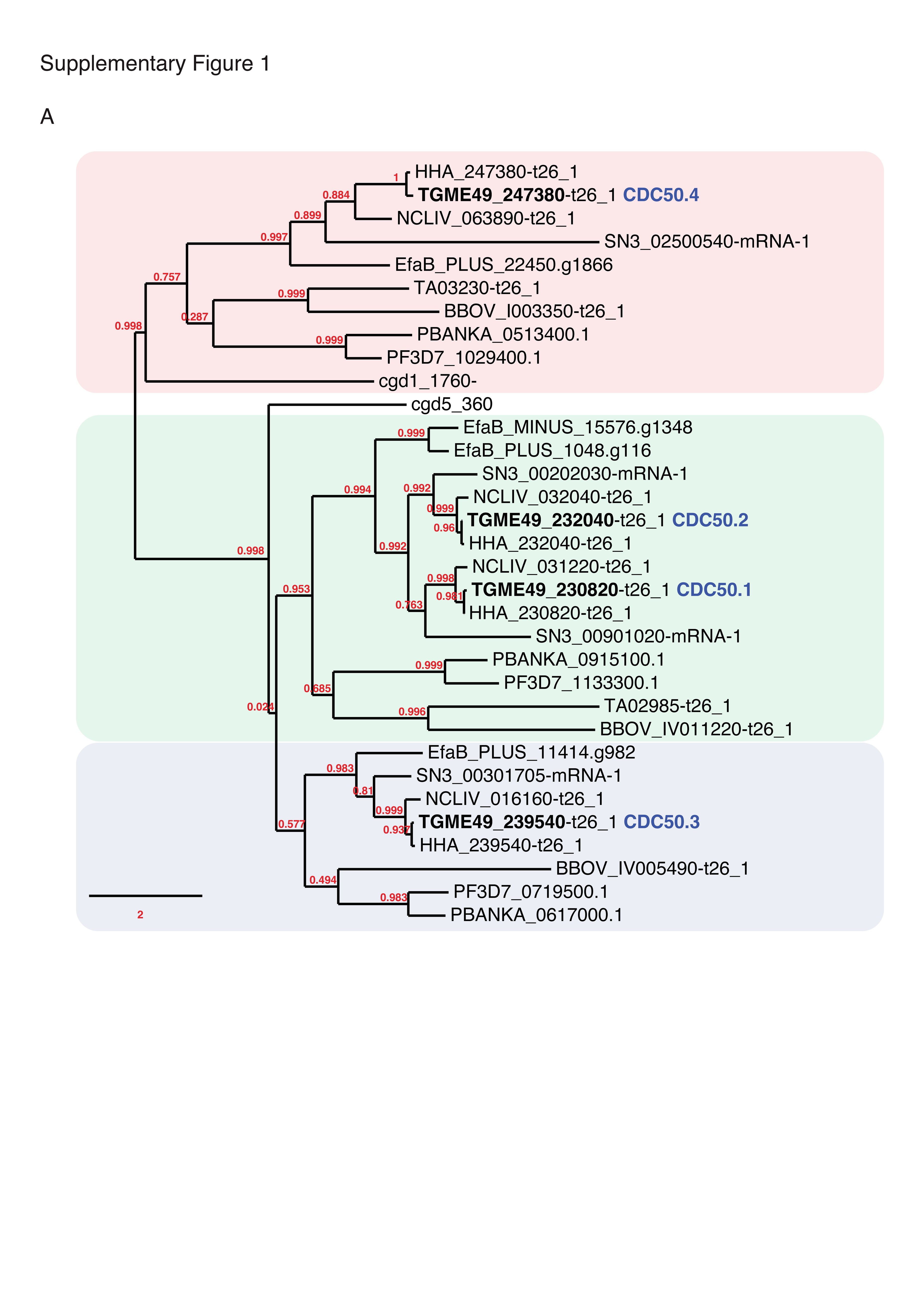

Supplement: S1 Fig — (A) Apicomplexan CDC50s cluster into 2 phylogenetic groups. An unrooted maximum likelihood tree of apicomplexan ASPs was generated using PhyML v3.0, using WAG model of amino acids substitution with NNI topology search, based on an amino acid alignment by MUSCLE. The genes are represented by the EuPathDB accession numbers. Node support values are indicated. (TIF) [file ppat.1010438.s001.tif]

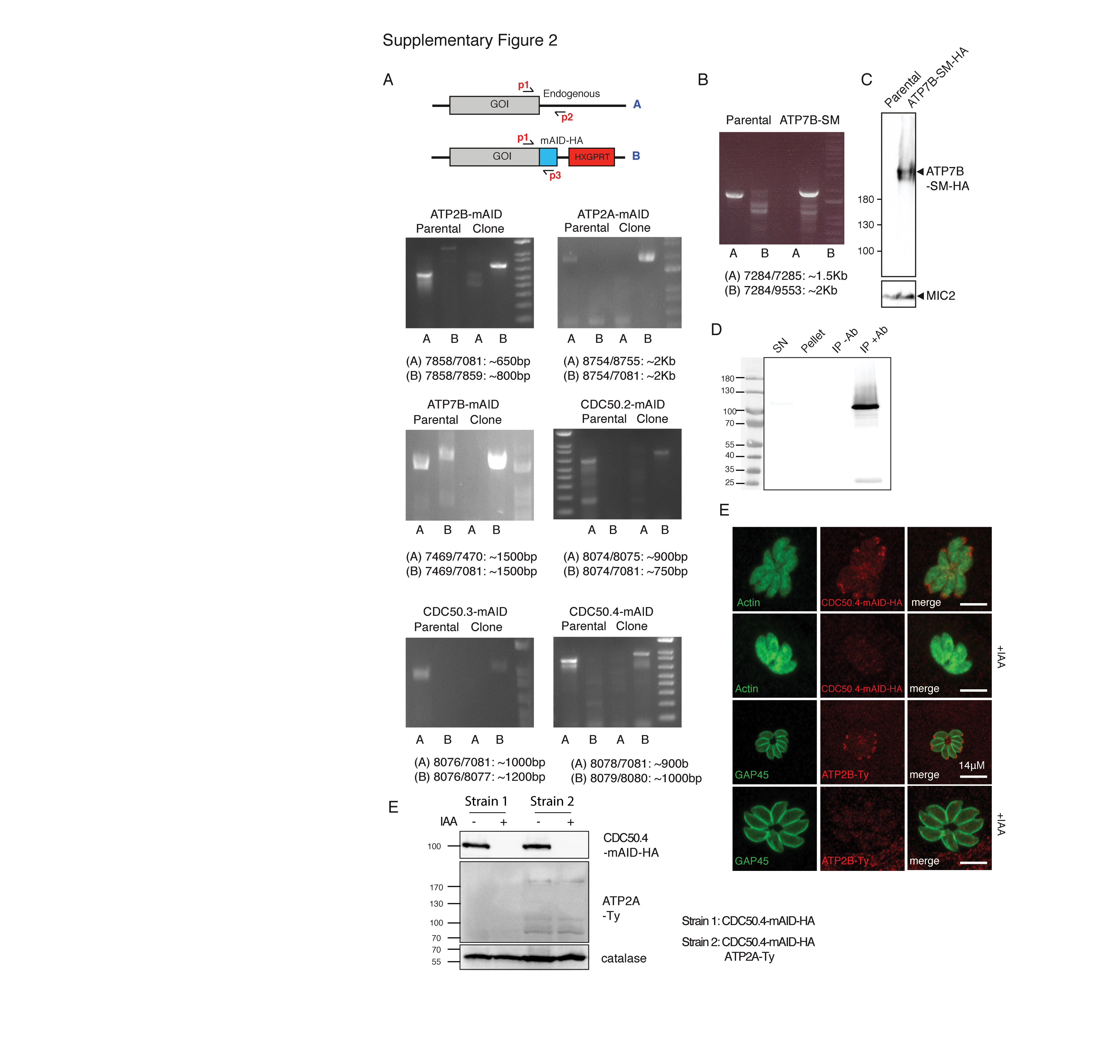

Supplement: S2 Fig — (A) PCR demonstrates correct integration of ATP2A, ATP2B and ATP7B and CDC50.2–4 mAID. Primers used are listed in S2 Table. (B) PCR demonstrates correct integration of ATP7B-SM-HA. Primers used are listed in S2 Table. (C) Immunoblot of lysates from RH parental and ATP7B-SM-HA parasites. HA antibodies were used to detect tagged ATP7B. MIC2: loading control. (D) Western blot showing enrichment of CDC50.4-mAID-HA upon immunoprecipitation. Anti-HA antibodies were used to detect CDC50.4-mAID-HA in the different fractions. S: soluble fraction (input), P: pellet fraction. (E) IFA of intracellular RH ATP2B-Ty/CDC50.4-mAID-HA parasites with or without IAA. The scale bars for the immunofluorescence images are 7μM, unless otherwise indicated. (F) Western blot of lysates ATP2A-Ty/CDC50.4-mAID-HA parasites treated with or without IAA for 24 hours. Catalase: loading control. (TIF) [file ppat.1010438.s002.tif]

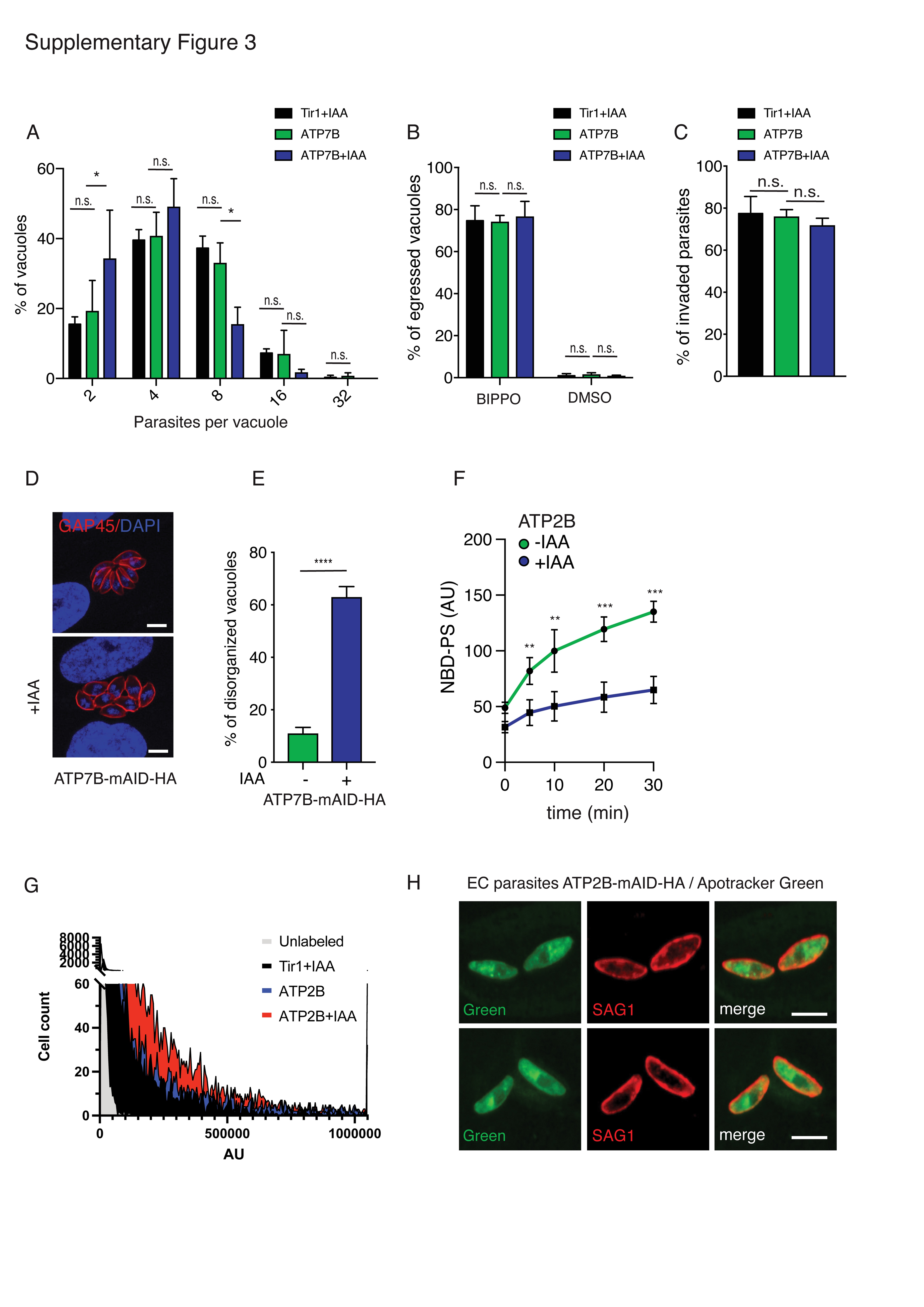

Supplement: S3 Fig — (A) Parasites lacking ATP7B display a delay in intracellular replication. Error bars represent ±SD from three independent experiments. (B) Egress assay of Tir1 parental strain and ATP7B-mAID-HA parasites grown for 30hs treated with or without IAA. Egress was induced with BIPPO or DMSO for 7 minutes. Percentage of egressed vacuoles is shown as means+/- SD of 3 independent replicates. (C) Invasion assay of Tir1 parental strain and ATP7B-mAID-HA parasites treated with or without IAA for 24 hours. Data represents mean +/- SD. (D) Representative images of vacuole organization in parasites depleted (or not) of ATP7B. Quantification is shown in (E). (F) Flow cytometry measurement of residual fluorescence upon addition of DPX to NBD-PS incubated extracellular ATP2B-mAID in intracellular buffer. Parasites were mechanically released from intracellular condition to avoid activation of egress signalling. (G-H) Histograms corresponding to one experiment of Annexin V binding to parasites are shown in (G) and representative images in (H). The scale bars for the immunofluorescence images are 7μM, unless otherwise indicated. (TIF) [file ppat.1010438.s003.tif]
