# Supplementary material for: Toxoplasma gondii phosphatidylserine flippase complex ATP2B-CDC50.4 critically participates in microneme exocytosis
Source: PLoS Pathog. 2022 Mar 24;18(3):e1010438. doi: 10.1371/journal.ppat.1010438 (PMC8982854; doi:10.1371/journal.ppat.1010438)
Supplement: Attachment — Submitted filename: Flippases_Rebuttal_17.11.21.docx [file ppat.1010438.s006.docx]

**Editor**

As you will see, all three reviewers are excited by the study and agree that it makes an important contribution to understanding of the role of P4-ATPase flippases in phospholipid asymmetry, microneme discharge and host cell invasion by Toxoplasma. However, the reviewers also share a number of substantial concerns with the manuscript in its current form. In particular, they agree that not all the conclusions are currently supported by the experimental data. Areas in which additional experimental evidence is requested include the suggestion that PS binds directly and specifically to DOC2.1. Examining the effects of ATP2B knockdown on DOC2.1 localisation would also inform the major conclusions, as would analysis of the effects of CDC50.4 knockdown on subcellular localisation of ATP2A and ATP2B. An examination of whether PS flipping occurs in intracellular parasites would also address the proposed link with egress. Whether these P4-ATPases can flip phospholipids other than PS has not been examined, although we do not feel this is an essential requirement. More detailed analysis of the calcium dependency of DOC2.1-PS binding is warranted. The inclusion of statistical analysis of the quantitative data (including full details in the Methods section of the statistical methods used), as well as information on numbers of experimental replicates, is essential. Finally, Figure 6 in particular requires attention and corrections, whilst a number of textual changes and corrections are requested throughout the manuscript that we agree in most cases would help to clarify the narrative.

**Reviewer #1**

The manuscript *'Toxoplasma gondii* phosphatidylserine flippase complex ATP2B-CDC50.4 critically participates in microneme exocytosis' presents some nice cell biological work utilising reverse genetics to detail the role of flippases in the asexual cycle of Toxoplasma. The authors identify P4-ATPase flippase proteins and their partner CDC50 chaperones and identify a potential, novel and essential role for an ATP2B-CDC50.4 complex in microneme secretion required for host cell invasion. Overall, the work present is well designed and the conclusions are well supported by the data presented.

**Reviewer #2**

The work by Bisio and colleagues provides important insights into P4-ATPases in Toxoplasma gondii - a class of enzyme that is involved in flipping phospholipids across membrane bilayers. Given previous findings that have shown that phosphatidic acid is important for mediating egress, this piece of work looks into the involvement of several P4-ATPases and their CDC50 chaperones, focusing mainly on egress and invasion. Epitope tagging and immunoprecipitation revealed that two ATPases, ATP2A and ATP2B, interact with the chaperone CDC50.4. Using the conditional auxin degradation system, Bisio et al reveal that CDC50.4 is important for ATP2B stability, but does not affect another P4-Type ATPases, GC. Using this system the authors go on to reveal that conditional knockdown of CDC50.4 and ATP2B leads to egress and invasion defects as well as a defect in microneme secretion. On the other hand, ATP7B knockdown leads to a disruption of intracellular growth and vacuole organisation, while disruption of ATP2A shows no defect at all.

Further characterisation of ATP2B and CDC50.4 revealed that ATP2B is involved in flipping phosphatidyl serine from the outer to the inner leaflet of the plasma membrane and that disruption of ATP2B leads to an accumulation of PS on the surface of tachyzoites. Next the authors take a side step and investigate the role of Doc2.1, a C2 containing protein involved in mediating microneme secretion, in sensing PS. The authors show that this protein does indeed bind to PS in a calcium independent manner. Overall these findings are important and show that regulating PS asymmetry is important in regulating processes such as egress and invasion and will be of great value to the Toxoplasma field. Despite these important findings, the manuscript in its current state requires significant improvement and amendments for publication. Most importantly: more experiments are required for ATP2A and CDC50.4 given that these are the major focus of this study, while the Doc2.1 data feels a bit like an add-on.

**Reviewer #3**

This work studies the role of a Toxoplasma gondii complex formed between the predicted flippase ATP2B with the cell division control protein (CDC) 50.4 for the flipping and asymmetric distribution of phosphatidylserine, which may act as lipid mediator for organelle fusion and microneme exocytosis. Authors first identified and localized putative flippases and CDC50s in T. gondii. They did this by C-terminal tagging of the respective proteins. A number of them localized to the apical end of the parasite. They decided to focus on CDC50.4 because of its predicted essentiality and conservation across the apicomplexans. They first demonstrated the interaction of CDC50.4 with ATP4A and ATP4B by immunoprecipitation coupled with mass spectrometry analysis. They tagged ATP2B in the CDC50.4-AID-HA background for co-localization studies. To study the function of these proteins they tagged them with the auxin-inducible degron (mAID) for downregulation. Downregulation of CDC50.4 led to degradation of ATP2B as shown by western blot analysis. They did not check for ATP4A. They next determined that the complex between ATP2B-CDC50.4 and also ATP7B were critical for T. gondii growth. They examined every step of the lytic cycle: invasion, egress and replication. Depletion of ATP2B or CDC50.4 caused impairment in invasion and egress but did not alter intracellular replication. Microneme secretion of extracellular parasites was reduced upon downregulation of either ATP2B or CDC50.4. No motility assays were shown. They showed that both proteins localize to the parasite membrane using a protease protection assay in non-permeabilized parasites. They measured flippase activity in situ with PS analogues, which was dependent on the presence of ATP2B at the PM. They also used a genetically encoded lactadherin C2 domain fused to GFP, known to bind PS. No controls for specific labeling of PS were shown. In conclusion authors claim to have identified a PS flippase that contributes to motility, invasion and egress. The authors claim that DOC2.1, a previously described key egress and invasion factor, senses changes in cytosolic calcium in intracellular parasites and may be the plausible sensor of PS at the inner leaflet of the plasma membrane. The work has some weaknesses concerning statistical analysis of the data and only partial information on the number of biological replicates for each experiment. Some statements are not supported by the data presented like the Ca2+ sensing of DOC2.1 or the motility defects of the mutants. Some improvements are suggested, and more explanations and discussion of results obtained are needed.

**Responses to the Editor and Reviewers’ comments**

**Liposome binding DOC2.1**

The three reviewers were aligned in raising concerns about the liposome binding for DOC2.1 and we fully agreed with them that further characterization is needed to support our claims.

Reviewer#1 is correct that the data shown were not implying any direct binding of DOC2.1 to PS. In the original manuscript we indicated “…. *PS might be recognized by DOC2.1, which is capable of, directly or indirectly, binding PS independently of calcium.”*

Importantly, the assessment of direct interaction between DOC2.1 and PS would require recombinant protein production and purification and our efforts to date were unsuccessful in producing the full-length protein (over 200KDa) in heterologous systems. Moreover, we also failed to produce soluble single or double C2 domains of the protein in *E. coli.* Taking all of this into account, we believe that the study of DOC2.1, which likely possess pleiotropic functions, would require a much-in-depth investigations that are in our view beyond the scope of this manuscript. In the contest of this manuscript, the objective was to stand out that DOC2.1 (directly or indirectly through other soluble components of the parasite cytoplasm) has a greater tendency to bind liposomes containing PS, which is a phospholipid enriched at the inner leaflet of the plasma membrane. This might contribute to the mechanism of fusion of micronemes with the plasma membrane for exocytosis, similarly to well-studied mechanisms proposed in model organisms (1-3).

While we have no reasons to assume that the binding of DOC2.1 to liposomes containing PS doesn’t hold true, we have experienced difficulties in performing these experiments in a reproducible manner during the revision. Particularly, detection of the protein by western blot has been problematic. It is important to stand out that these liposome binding experiments are not a trivial experiment considering the extremely low abundance of DOC2.1 (which even precluded its subcellular localization determination until this manuscript) and its binding to membranes, which also leads to a loose of 1/2 to 2/3 of the protein in the insoluble fraction. Furthermore, the high molecular weight of the protein makes also difficult the transfer for western blotting. Given that our efforts led to inconclusive results we have opted to remove the liposome binding assay from the manuscript and only speculatively discuss the possibility that DOC2.1 might bind to PS.

**Part II – Major Issues: Key Experiments Required for Acceptance**

**Reviewer #1**

The work characterising the essentiality of identified CDC50 and P4-ATPase proteins has been carried out well. This has been recently published in an overlapping study (Ref 30) and therefore there should be further discussion of this published work included in the manuscript.

We agree with the reviewer. We have now extensively remodeled the discussion and commented in more detail the findings reported in the Reference: *Chen K, Gunay-Esiyok O, Klingeberg M, Marquardt S, Pomorski TG, Gupta N. Aminoglycerophospholipid flipping and P4-ATPases in Toxoplasma gondii. J Biol Chem. 2021:100315.*

However, the present study adds a detailed view of the role of these proteins at specific stages of the asexual cycle. The authors clearly demonstrate that ATP2B and CDC50.4 forms an essential complex and in some very nice experimental work, show that this complex translocates phosphatidylserine (PS). They then go on to mechanistically characterise the role of the ATP2B-CDC50.4 complex and PS translocation. The authors indicate that PS translocation by ATP2B-CDC50.4 is required for microneme secretion, and this is carried out by DOC2.1 binding of PS. Whilst this is a nice conclusion, the data presented to support the premise of DOC2.1-PS binding is weak and requires more substantial affirmation. Given that liposome-binding experiments were performed with parasite lysate, it cannot be discounted that DOC2.1 binding is not direct but mediated by another protein factor, the text should be amended to reflect this.

Please refer to the liposome binding section presented at the beginning of the response to reviewers. Moreover, beside the postulated connection between PS and DOC2.1, we have also indicated the possibility of other components, including changes in membrane tension, membrane curvature or other membrane associated signaling molecules/proteins to be influenced by modification in plasma membrane asymmetric distribution. Indeed, it is likely that the phenotype observed upon ATP2B or CDC50.4 knockdown could be explained by a combination of several these factors.

Added to the discussion: “*The disruption of this homeostasis might lead to overall changes in plasma membrane tension, curvature and could also affect the activity of important signalling components (i.e. GC (8-11), PKG (14), PI-PLC or DGK1 (21)), explaining the phenotype associated with the depletion of ATP2B-CDC50.4 complex”.*

Additionally, since PS is an anionic lipid, it is possible that liposome binding of DOC2.1 from lysate is simply due to charge-charge interaction. The group's previous work has demonstrated the production of the anionic lipid phosphatidic acid (PA) at the plasma membrane preceding egress, which is required for microneme secretion. Given this, the authors should repeat the sedimentation experiment of tagged DOC2.1 using liposomes containing PA to rule out that interaction is not PS specific but due to binding of anionic charge.

Please see comments about the liposome binding at the beginning of the response to reviewers.

Furthermore, it would add more support to the hypothesis of DOC2.1-PS interaction if the study could visualise the localisation of tagged DOC2.1 in egressed parasites with and without knock-down of ATP2B. Presumably, the localisation of DOC2.1 would become increased in plasma membrane proximity which would likely be lacking upon ATP2B knock-down.

The localization of DOC2.1 in extracellular parasites was examined and no significant changes were observed to the cytoplasmic localization shown in intracellular parasites **(new Figure 6C).** We also induced microneme secretion using BIPPO or calcium ionophore without noticeable changes in DOC2.1 localization. Depletion of ATP2B did not alter DOC2.1either.

The authors outline in Figure 5A and 5B that invasion and egress are impaired in ATP2B and CDC50.4 knock-down parasites. However, looking at the data it seems that invasion is similarly impaired to the extent of the lack of egress. Can the authors rule out that invasion occurs normally, and the reduction seen is merely a result of the reduction in ATP2B and CDC50.4 knock-down in parasite egress?

To perform invasion assays, we routinely syringe lyse the infected cells to avoid confounding effects resulting from impairment in egress. In consequence, we are confident that the observed invasion defect upon ATP2B and CDC50.4 depletion is disconnected form defect in egress.

The authors suggest that there is no significant effect on microneme secretion by ATP2A knock-down, however, in Figure 5I there appears to be a ~50% reduction in MIC2 secretion in ATP2A knock-down parasites in the presence of BIPPO. Could the authors comment on this? Is this reproducible? If so, this needs to be acknowledged in the main text.

We thank the reviewer for pointing this out. We have now re-analyzed the data and the quantifications are discussed in the text. Of note, despite some mild level of reduction in MIC2 secretion, these changes are not statistically significant **(new Figure 4I).**

Figure 6H is hard to interpret. It is unclear what the significance value relates to. Additionally, it is difficult to clearly see the increase in annexin V binding in ATP2B knock-down tachyzoites. Perhaps displaying the data as a box plot would increase clarity.

We have followed the reviewer recommendation to display this data with a bar plot with the mean changes of annexin V staining in the triplicate experiments **(new Figure 5J),** and also included example of a histogram and image of fluorescence in the supplementary materials as suggested by reviewers # 2 and 3 **(new** **Figures S3G and S3H).**

Figure 6I shows the potential localisation of PS in WT parasites, however was the visualisation of internal PS attempted in ATP2B or CDC50.4 knock-down parasites? If so, was there any difference? If not, a comment should be included in the manuscript.

We have now included this data in the manuscript **(new Figure 5H).** No major changes were observed in the localization of the probe upon depletion of ATP2B. This result is not unexpected since PS is the most abundant anionic phospholipid in eukaryotic membranes (accounting up to 10% of the total cellular lipids) and it is highly concentrated at the inner leaflet of the plasma membrane (4). Concordantly, due to the high affinity of LacC2 to PS (5), low concentration of PS would be sufficient for its binding and re-localization. It is for this reason, that the labeling by Annexin V is a better marker to observe changes by downregulation of ATP2B, since levels of PS at the outer leaflet of the plasma membrane are low in wild type conditions.

Lines 87-89 Although there are references cited for the statement regarding phosphodiesterases (PDEs): '...presumably regulated by PKA-C1 and cAMP...', it leaves the reader wondering why the authors presume this. The statement either should be omitted or modified to outline briefly any evidence that regulation of TgPDEs may be by cAMP/PKA.

We agree with the reviewer. We have now introduced the following sentence in the introduction:

*“The level of cGMP is tightly controlled by phosphodiesterases, which differential phosphorylation state upon inactivation of the protein kinase A (PKA-C1) indicates that are presumably regulated by cyclic adenosine monophosphate (cAMP) levels (12, 13)”.*

Line 186, this results section would benefit from brief mention of some of the other proteins identified in the pull down/MS experiment shown in Figure 2.

We have now added the following sentences in the result section *“Other proteins identified in the pull down are presumably contaminants, since they correspond to highly abundant proteins or are predicted to perform non-related functions”.*

Line 220, should explain that proteins exposed at the surface of the PM are susceptible to the protease (rather than this just indicating PM localisation).

We have now added the following sentences in the text: *“Proteins exposed to the outer surface of the plasma membrane are susceptible to cleavage by proteases. Concomitantly, the disappearance of the full length ATP2A, ATP2B and CDC50.4 upon protease treatment demonstrates that these complexes localize to the plasma membrane of the parasite*”

Line 296-298, it should be clarified that this statement refers to P. yoelii erythrocytic stages (Ref 52) rather than 'Plasmodium species' in general.

Thanks for pointing this out. We have now clarified as follow: “*Plasmodium* CDC50A and CDC50B belong to the same phylogenetic subgroup that T. gondii CDC50.1 and CDC50.2, and would be good candidates to bind GCα Conflictingly, neither of these proteins are essential for *Plasmodium yoelii* erythrocytic stages (PY17X_0619700 and PY17X_0916600) (57) and, despite discrepancies with a genome wide screening on *P. falciparum* (58), would suggest a possible functional redundancy between CDC50A and CDC50B”.

The section describing the results shown in Figure 5 would benefit from briefly mentioning and explaining the use of the PDE inhibitor BIPPO. I note that its use is mentioned but not explained in the figure legend.

Info about BIPPO has been added as followed: “*Egress was induced with BIPPO (PDE inhibitor which induces the accumulation of cGMP in the cell) or DMSO for 7 minutes”.*

Figure 7, what is the likely explanation for the fact that mutation of the two aspartates in DOC2.1 causes reduced fitness but not invasion or microneme secretion? Could it e.g. mean that these residues (and perhaps calcium binding) have a role in parasite growth, but not at the stage of invasion/microneme secretion?

The reduced fitness of the DOC2.1 mutant is presumably due to an impaired egress and not to an intracellular growth defect. In order to confirm this, intracellular growth assays of the mutants have been included **(new Figure S4E).** We hypothesize that the C2 domain of DOC2.1 (and the potential calcium binding associated to it) might be more important in intracellular parasites, where the machinery for microneme secretion is strongly inhibited. During egress, some of this inhibition might be lifted, as previously suggested (6), with the calcium binding capacity of DOC2.1 becoming dispensable. We have now deeply reorganized the discussion in order to make this point clearer to the reader.

Line 277, were different concentrations of 'calcium' tested to decide on the use of 0.1 mM? If not, can it be concluded from this ~30% reduction in bound protein that binding is independent of calcium?

Given the technical difficulties we have opted for removing the liposome binding section. See comments at the beginning of the response to reviewers.

**Reviewer #2**

• Line 173: in this section, the authors focus on ATP2B but not ATP2A and it is unclear why the experiments were done solely on ATP2B, despite evidence of ATP2A also binding to CDC50.4. It would be interesting to see if ATP2A mislocalises when CDC50.4 is knocked down or whether there are other chaperones that can compensate for loss of CDC50.4.

We agree with the reviewer. We originally focused on ATP2B due to its importance for the fitness of the parasites. We have now included experiments demonstrating that in the absence of CDC50.4, similarly to ATP2B, ATP2A also reduces its concentration in the parasite **(new Figure 2E).**

• Line 183: the authors demonstrate that knockdown of CDC50.4 leads to a knockdown of ATP2B levels but they do not show an IFA of localisation of ATP2B-Ty following treatment with IAA. Does the remaining protein localise to the apical end of the parasite or is the localisation affected?

We have now included IFA data that compares the signal of ATP2B upon downregulation of CDC50.4 **(new Figure S2E).** Importantly, level of ATP2B by IFA in absence of CDC50.4 is not high enough to assess possible change in localization upon CDC50.4 depletion.

• Line 189: the authors offer no explanation of the lines that were generated. Please add a sentence explaining to the readers that the proteins of interest were fused to a mAID tag

Apologies for the insufficient information. We have now clarified it follows: “*The three P-type IV ATPases (ATP2A, ATP2B and ATP7A) and CDC50s (CDC50.2, CDC50.3 and CDC50.4), were C-terminally fused to the auxin-inducible degron (mAID) at the endogenous locus (14) and efficiently depleted upon addition of 3-indoleacetic acid (IAA) as confirmed by western blot”.*

• Line 208 & Figure 5D: the authors state in Line 208 that they assessed microneme secretion by stimulating with ethanol, however the figure legend of Figure 5D states that the parasites were stimulated with BIPPO. Can the authors correct either the legend or the main text since it is unclear which stimulant was used.

Thanks for pointing out this error. We have fixed this mistake in this text to accurately describe the experiment performed.

• Line 223: The authors decide to focus solely on PS experiments on ATP2A and ATP2B. Given their proven ability to do these assays with other phospholipids (PA and PC in the Bisio et al 2019 paper) and also given that they later state in the discussion that these ATPases may be involved in flipping PE, the authors should do these flipping experiments with PA, PC and PE as these would be highly informative and reduce the some speculation in the discussion section. Despite ATP7B not being involved in egress or invasion, it would also be useful for the greater Toxoplasmosis field and our understanding of Toxoplasma biology to know whether this ATPase is flipping any phospholipids, so it would also be important to include these experiments too since these lines are available.

We agree with the reviewer that the study of the flipping of other phospholipids would be of great interest. We had originally invested significant amount of time in the assessment of flipping of PA, PC and PE. Particularly, the low levels of flipping of PA and PC, as we have previously reported, made the analysis difficult. PE flipping has also been assessed but we faced technical difficulties in the quantifications. In our experience, PS has been the only phospholipid for which robust quantifications of flipping can be easily obtained. Concordantly, we have only included this data in the manuscript. Importantly, despite the interest on the general phospholipid biology, the focus of this manuscript is on the importance and homeostasis of PS and egress. For these reasons, we believe that the study of flip-flop of other phospholipids is out of the scope of this manuscript and will be addressed in future studies.

• Line 227: the authors look at PS flipping in extracellular parasites. It would also be important to look at whether this flipping occurs in intracellular parasites i.e. by performing this experiment on parasites lysed in ENDO buffer. This is an important experiment to do to support the author's model of PS accumulating on the inner leaflet prior to egress, since if this flipping is also occurring in intracellular parasites without any stimulation then in theory there shouldn't be any PS flipping onto the inner leaflet.

We have now included flippase assays performed as suggested by the reviewer (**new Figure S3F).** No differences were observed compared to the previous data shown on extracellular conditions. This suggests that ATP2B is active in intracellular conditions and this process is also impaired upon downregulation of the protein inside of the host cell. Importantly, it does not imply that an increase in PS flipping or concentration of PS at the inner membrane is directly linked to egress. To be precise, plasma membrane asymmetry is essential for the cell biology of any cell of any organism, and flipping of PS would need to be maintained across the entire lytic cycle of the parasite for survival. However, what is shown here is that the immediate consequence of the dysregulation of PS asymmetry at the plasma membrane is on egress, invasion and microneme secretion. We have edited the discussion to emphasize this point.

• Line 234: the authors provide little commentary on the results from the Lact-C2 experiment - is there a difference in binding between the ATP2BmAID mutants +/-IAA? It also appears they have mixed up the Figures since Figure 6H is the annexin experiment mentioned further down and figure 6I is the Lact-C2 experiment. Furthermore in Figure 6I it is unclear which parasites are shown in the image and there should be images comparing ATP2B parasite - and + IAA. Not to mention that this subfigure (6I) has no figure legend.

We have now included this data in the manuscript **(new Figure 5H).** No major changes were observed in the localization of the probe upon depletion of ATP2B. This result is not unexpected since PS is the most abundant anionic phospholipid in eukaryotic membranes (accounting up to 10% of the total cellular lipids) and it is highly concentrated at the inner leaflet of the plasma membrane (4). Concordantly, due to the high affinity of LacC2 to PS (5), low concentration of PS would be sufficient for its binding and re-localization. It is for this reason, that the labeling by Annexin V is a better marker to observe changes by downregulation of ATP2B, since levels of PS at the outer leaflet of the plasma membrane are low in wild type conditions. The figure legend and text have been updated accordingly.

• Line 235: the authors state that ATP2B knockdown parasites are unable PS asymmetric distribution - are there images to support this? The graph shown (Figure 6H shows that there is maybe more binding of Annexin V) but it would aid the reader if images of these parasites are shown. Is this increased accumulation of Annexin at the apical tip where ATP2B is localised to? Please provide these images as this is critical information that the reader should be able to see.

We have now included pictures of staining with annexin V in **(new Figure S3H).** Importantly, the staining of annexin V is not exclusively restricted to the periphery. This phenomenon has been constantly reported in the literature and depends on PS exposure at the outer leaflet of the plasma membrane (7-10). Consequently, more precise localization of PS cannot be performed by this method.

• Line 273: have the sedimentation experiments been done with the mutant versions of Doc2.1? If not the authors should perform these experiments since there appears to be a difference in the binding of PS + and - Ca2+ (despite it not being the pattern that the authors expected).

See comments in regard to the liposome binding section at the beginning of the response to reviewers. Due to the technical challenge and insufficient reproducibility, we opted to remove the data and the corresponding claim.

• Line 298: the authors reference the Jian et al paper from 2020 as the source for CDC50A and CDC50B not being essential for Plasmodium - however these experiments are nowhere to be found in this source. The authors should reference the correct source here. As far as I am aware there is only evidence of CDC50A not being essential in P. yoelii (Gao et al 2018) but I couldn't find one for CDC50B

Indeed, the information is not easy to access in the paper cited since the proteins are mentioned only by their accession number. We have clarified this point as followed:

“*Plasmodium* CDC50A and CDC50B belong to the same phylogenetic subgroup that T. gondii CDC50.1 and CDC50.2, and would be good candidates to bind GCα. Conflictingly, neither of these proteins are essential for *Plasmodium yoelii* erythrocytic stages (PY17X_0619700 and PY17X_0916600) (57) and, despite discrepancies with a genome wide screening on *P. falciparum* (58), would suggest a possible functional redundancy between CDC50A and CDC50B”.

• Line 298: after checking PlasmoDB and looking at the mutagenesis screen by John Adams lab, it appears that CDC50B is essential and CDC50C is important for asexual growth. The authors state here that CDC50B is not essential and do not mention CDC50C in the discussion

We thank the reviewer for sharing the information. We have pondered these discrepancies previously but, since (Jian et al 2020) provides data specifically generated for these genes rather than a genome wide screening, we had opted to prioritize those data. However, given the differences in Plasmodium species we agree that it is important to be more comprehensive. We have now commented on the discrepancies on the data as shown in the previous point.

• Line 353: the authors mention the recent FER1 pre-print, but this text seems out of place and needs more explanation or to be rewritten to link it to the rest of the discussion

We have now rephrased the discussion as follows: “i*n this context, the notion of primed micronemes has previously being suggested (37, 63). Important, a recent study reports that the overexpression of FER1 led to an accumulation of apical localized micronemes and an increase of constitutive secretion of these organelles (37) suggesting that FER1 would be a component of the priming machinery for microneme secretion”.*

• The discussion needs substantial improvements. At times it is difficult to follow the order and reasoning behind the information included. The authors should re-write this and also include summaries of their experiments. For example there is no mention of ATP7B or any of the Doc2.1 experiments which constitute a small yet significant part of the paper.

The discussion has been remodeled as recommended by the reviewer. We hope the new version reads more clearly and covers all results presented in the manuscript.

• Line 595: the authors should change "(D-F)" to (D-G) since figure G is also a part of this series of experiments.

Corrected

• Line 597: the authors state in the legend that figure 6G represents the staining oft he plasma membrane and internal organs of the parasite with LactC2 but the actual figure doesn't correspond to this. Can the authors please add the appropriate image and fix the legend.

Thanks. This mistake has been fixed.

• Line 599: in Figure 6 there is a subfigure 6I, however there is no reference to this subfigure in the figure legend. Please add a description of this image explaining what the parasite line is.

Corrected

• Figure 5: no statistical analysis for any of the graphs after figure 4 are provided. The authors should provide this and if there is a statistically significant difference in the levels of PS binding of Doc2.1 + and - Ca2+ (which it looks like there is), then this should be commented on in the manuscript.

Statistical analysis has now been included in all figures.

• Throughout the manuscript no statistical tests were performed. I would expect the appropriate stats tests to be performed for all graphs without exception. Without this data, the reader is missing crucial bits of information to make their own judgements on the data.

We agree with the reviewer. Statistical analysis has been included in all figures.

• Methods section: there is no methods section for the AnnexinV binding experiments - please add this.

The materials and methods section have been updated to incorporate methodological detail about the experiment.

**Reviewer #3**

1. Figure 2E shows a lot of background with the GC-TY. Any explanation for that?

We agree with the reviewer and apologize for the quality of the images. All flippases, included the GC, are polytopic membrane proteins and are expressed in low quantities in the cell. Concomitantly, their detection by IFA is challenging. We have now recorded new data and improve the quality of the images presented (**new Figure 2F)**.

2. The IFA image shown for the downregulation of CDC50.4 appears to show some effect on the expression/distribution of GC and it concentrates in the residual body?. This result needs quantification and a better description in the legend

Of note, the levels GC at the apical and residual body are not homogeneous and vary from one parasitophorous vacuole to another. For this reason, measuring subtle changes in its localization is not an easy task. We have nevertheless made such quantifications comparing the intensity of fluorescence at the apical or the residual body of the parasite (**new Figure 2G)**. No significant changes in the ration of intensity of fluorescence apical versus basal were observed.

3. There are no attempts to localize ATP2B in the CDC50.4 knock downs.

We have now included IFA comparing the signal of ATP2B upon downregulation of CDC50.4 **(new Figure S2E).** Importantly, levels of ATP2B in absence of CDC50.4 are not high enough to allow it a detection by IFA, so we cannot address the question if changes in localization are also happening upon CDC50.4 depletion

4. Figure 3C: the plaques for CDC50s are strange. Either there are no plaques in the image shown or they are not fully lysed. It will be good to explain what was measured and quantified for part D. Indicate in the legend the number of biological replicates.

Indeed, as deducted by the reviewer the plaques were not fully lysed, making it difficult to visualize. In order to improve the quality of the images displayed, we have repeated the plaque assays experiments (**new Figure 3C).**

5. Figure 4 presents the phenotype of ATP7B and is not clear how it fits in the story. Authors do not give an explanation of the disrupted rosette phenotype observed.

Arguably, we did not focus our attention on ATP7B in this study. The data presented are informative and useful for future research in the area. In consequence, we have move them in the supplementary materials **(new Figure S3).**

6. Figure 5, please indicate in the legend the number of biological replicates for all panels (some are missing) and show statistical significance analysis for the ones that are relevant.

The relevant information about number of biological replicates and statistical analysis is provided throughout the study.

7. Figure 6: The ATP2B-CDC50.4 complex localizes to the apical end or the plasma membrane? This should be consistent in the description of the results. The proteolysis experiment shows that the HA epitope is degraded but not necessarily the whole proteins.

We have now added the following sentences in the manuscript: “*Proteins exposed to the outer surface of the plasma membrane are susceptible to cleavage by proteases. Concomitantly, the disappearance of the full length ATP2A, ATP2B and CDC50.4 upon protease treatment demonstrates that these complexes localize to the plasma membrane of the parasite”*

8. Figure 6D-F: indicate the number of biological replicates and any statistical significance obtained in the legend. Labeling of panels need to be corrected in the figure which does not agree with the legend.

Number of biological replicates and statistical analysis has now been carefully reviewed throughout the manuscript.

9. Figure 6H: The flow cytometry charts could be shown as supplemental. The differences in the violin plots results are not clearly evident from the distribution. Need to detail in the legend, the number of independent replicates and explain more how the statistical analysis was done. The p value shown is for which comparison?

We have revised the way to display the data. We used a bar plot with the mean changes of annexin V staining in the triplicate experiments **(new Figure 5I),** and as example a histogram and image of fluorescence in the supplementary material **(new Figures S3F and S3G).**

10. Figure 6H and I are swapped in the text.

This mistake has been corrected.

11. Figure 6I: Some evidence of specific binding is needed. Is there a cell line depleted of PS that could serve as control for the binding of the lactadherin-GFP? Reference 59 generated mutants for PS synthesis although the PS content of the mutants was not clear, and it was a double mutant. Specificity could also be shown by transfecting parasites with the mutated version of the Lac-gfp gene as shown in reference 42.

To our knowledge, there is no parasite knockout made which has been demonstrated to reduce specifically the level of phosphatidylserine as the cell lines generated in reference 59 do not demonstrate clear reduction in PS. Instead, we have now included data demonstrating that mutations in the binding of PS by LacC2 impede its localization to the plasma membrane **(new Figure 5I).**

12. Figs 7 D and E need quantification of plaque sizes and statistical analysis

Quantification of plaque sizes has now been included.

13. Figure 7F needs statistical analysis and the legend needs to indicate the number of biological replicates for G.

Statistical analysis and number of replicates have been included in all figures.

14. Figure 7H needs quantification and statistical analysis. Indicate the number of replicates. Could this part be re-organized with the legends at the top? Also, separate the bottom panel since it is very confusing as presented.

Quantification has now been included and the figure has been reorganized as recommended.

15. Figure 8: Legend needs to indicate the number of biological replicates. The data in B needs statistical analysis.

Given the technical difficulties we have opted for removing the liposome binding section. See comments at the beginning of the response to reviewers.

16. Figure 8: Authors show that DOC2.1 binding to PS liposomes is independent of calcium. The concentration of free calcium is difficult to predict from the information provided. EDTA mainly chelates Mg2+ and EGTA would be more appropriate. The concentration of Ca2+ of the lysate could be high considering that Ca2+ bound to proteins would be released during lysis of the cells. 0.1 and 1 mM EGTA should be tested to be sure that calcium is not relevant. In addition, there is no information of the Ca2+ binding affinity of DOC2.1

See comments on liposome binding section at the beginning of the response to reviewers.

17. Abstract says that DOC2.1 senses changes in cytosolic Ca and that is a sensor of PS at the inner leaflet of the PM. The data only shows that mutating putative calcium binding domains results in growth defects due to egress defect. There is no evidence for Calcium binding of DOC2.1 so the mutation could impact other aspects of the protein. May be increases in cytosolic Ca affects the localization of DOC2.1 making it more defined? This would be interesting to explore.

We agree with the reviewer. Given the changes made to content of the manuscript we have now changed the abstract as follows:

*“DOC2.1, a previously described key egress and invasion factor, is shown here to be affected in its function in egress upon mutation on residues putatively involved in calcium binding”*

We have also assessed the localization of DOC2.1 in extracellular parasites in presence or absence of calcium ionophore as suggested by reviewers # 1 and 3. No changes in localization were observed as shown in the **new Figure 6C.**

The methods section does not have an explanation for the statistical analysis of the data.

The materials and methods section have been updated to incorporate methodological detail about the statistical analysis.

**Part III – Minor Issues: Editorial and Data Presentation Modifications**

**Reviewer #1**

Line 33, insert 'the' parasite.

Line 64, insert 'the' plasma membrane.

Line 67, Perhaps modify 'inner enriched gradient' which doesn't sound quite right. It reads well in line 133.

Line 68, delete 'the'.

Line 73, 79 should be 'phylum Apicomplexa'. Delete 'of'.

Line 82, 'stages'.

Line 89, define PKA as the cAMP-dependent protein kinase.

Line 90, delete 'G' so that PKG is defined correctly.

Line 96, Perhaps replace 'system' with 'motor'

Line 117, 'based on'.

Line 128, Perhaps replace 'participating' with 'involved' or 'strictly participates'.

Line 136-139 This section containing two presumptions would benefit from a re-write and isn't a strong end to the Introduction.

Line 137, 'presumable' should be replaced by e.g. 'putative'.

Line 157, replace 'aroused' with 'arisen'

Line 175, 'focused'.

Line 177, e.g. delete 'an'.

Line 199, e.g. delete 'the'.

Line 200, replace 'every' with 'each of'.

Line 201, delete 'in repaired'.

Line 205, 'parasites'.

Line 217, 'of the complexes'.

Line 222, 'cell'.

Line 224-225, please re-write the last half of this sentence.

Line 225, e.g. 'A bulk time'.

Line 235, 'parasites'.

Line 236, 'the asymmetric PS distribution'.

Line 238, 'taken together these results indicate that...'.

Line 242, 'micronemes'.

Line 247, delete 'the'.

Line 251, Delete 'A'.

Line 293, specify that in P. yoelii it is GCβ

Line 294, 'groups with' would benefit from slight expansion.

Line 363, 'remained'?

Line 629, state here what calcium salt was used.

All minor issues have been addressed and corrected.

**Reviewer #2**

• Line 157: please change "aroused" to "have arisen"

• Line 166: please consider changing sentence to "Similar to GC and its partner CDC50.1, which have previously been found at the apical tip of the parasites (8), ATP2A, ATP2B, CDC50.2 and CDC50.4 are also found at the apical tip"

• Line 191: why do some of the western blots have a parental sample while some don't?

• Line 217: the authors provide no rationale behind the protease protection experiments. A short sentence here explaining the reasoning behind this or the line of thought would greatly aid the readers in following the story.

• Line 353: unclear what is meant by "tonic"

• The authors have size bars for all IFAs but they fail to state what size this bar corresponds to in any of the figure legends. Please include this

• The authors should consider merging Figures 7 & 8

• Figure 6: the scale bars of 6E should be amended to be the same as the scale bars the other figures

• Figure 6: the statistical analysis of Figure 6H is missing despite there being a p value

All minor issues have been addressed and corrected.

**Reviewer #3**

1. Tables S1 and S2 are mislabeled.

2. The links in Table S2 (should be S1) all lead to a "page not found"

3. Figure 2C needs markers

4. Although is not relevant to the story, the authors mention in the introduction that the signaling cascade starts with cGMP, which activates PKG, PI-PLC, IP3, calcium and CDPKs. However, it should also be taken into consideration that PIPLC needs calcium for activity, so the pathway may not be that linear and calcium could also be upstream to PIPLC.

All minor issues have been addressed and corrected.

**References**

1) Brouwer, I., Giniatullina, A., Laurens, N. *et al.* Direct quantitative detection of Doc2b-induced hemifusion in optically trapped membranes. *Nat Commun* **6,** 8387 (2015). <https://doi.org/10.1038/ncomms9387>
2) Orita, S., Sasaki, T., Naito, A., *et al.* Doc2: A Novel Brain Protein Having Two Repeated C2-like Domains. *Biochem and Biophysical Research Commun*, **206**, 2 (1995). <https://doi.org/10.1006/bbrc.1995.1062>.
3) Yao J, Gaffaney JD, Kwon SE, Chapman ER. Doc2 is a Ca2+ sensor required for asynchronous neurotransmitter release. *Cell*. 2011;147(3):666-77. [10.1016/j.cell.2011.09.046](https://dx.doi.org/10.1016%2Fj.cell.2011.09.046)
4) Kay, J.G., Fairn, G.D. Distribution, dynamics and functional roles of phosphatidylserine within the cell. *Cell Commun Signal* 17, 126 (2019). <https://doi.org/10.1186/s12964-019-0438-z>
5) Shao, C., Novakovic, V. A., Head, J. F., Seaton, B. A., & Gilbert, G. E. (2008). Crystal structure of lactadherin C2 domain at 1.7 A° resolution with mutational and computational analyses of its membrane-binding motif. Journal of Biological Chemistry, 283(11), 7230–7241. <https://doi.org/10.1074/jbc.M705195200>
6) Bisio H, Soldati-Favre D. Signaling Cascades Governing Entry into and Exit from Host Cells by Toxoplasma gondii. *Annu Rev Microbiol*. 2019;73:579-99. 10.1146/annurev-micro-020518-120235.
7) Miao, Q.; Bi, L.-L.; Li, X.; Miao, S.; Zhang, J.; Zhang, S.; Yang, Q.; Xie, Y.-H.; Zhang, J.; Wang, S.-W. Anticancer Effects of Bufalin on Human Hepatocellular Carcinoma HepG2 Cells: Roles of Apoptosis and Autophagy. Int. J. Mol. Sci. 2013, 14, 1370-1382. <https://doi.org/10.3390/ijms14011370>
8) Xian Nan Tang, Qing Wang, Maya A. Koike, Danye Cheng, Michael L. Goris, Francis G. Blankenberg, Midori A. Yenari. Monitoring the Protective Effects of Minocycline Treatment with Radiolabeled Annexin V in an Experimental Model of Focal Cerebral Ischemia. *Journal of Nuclear Medicine* Nov 2007, 48 (11) 1822-1828; DOI: 10.2967/jnumed.107.041335
9) Jin X, Fu W, Li D, et al. High Expression of LINC01268 is Positively Associated with Hepatocellular Carcinoma Progression via Regulating MAP3K7. Onco Targets Ther. 2021;14:1753-1769. Published 2021 Mar 8. doi:10.2147/OTT.S295814
10) Chen D, Su H, Li Y, Wu X, Li Y, Wei C, Shi D, Gao Y, Zhou Q, Wang Q, Jin X, Xie C. miR-20b and miR-125a promote tumorigenesis in radioresistant esophageal carcinoma cells. *Aging (Albany NY)*. 2021 Mar 10;13(7):9566-9581. doi: 10.18632/aging.202690.
