# Supplementary material for: Toxoplasma gondii phosphatidylserine flippase complex ATP2B-CDC50.4 critically participates in microneme exocytosis
Source: PLoS Pathog. 2022 Mar 24;18(3):e1010438. doi: 10.1371/journal.ppat.1010438 (PMC8982854; doi:10.1371/journal.ppat.1010438)
Supplement: Attachment — Submitted filename: rebuttal_2022.docx [file ppat.1010438.s007.docx]

Thank you very much for submitting your manuscript "Toxoplasma gondii phosphatidylserine flippase complex ATP2B-CDC50.4 critically participates in microneme exocytosis" for consideration at PLOS Pathogens. As with all papers reviewed by the journal, your manuscript was reviewed by members of the editorial board and by several independent reviewers. The reviewers appreciated the attention to an important topic. Based on the reviews, we are likely to accept this manuscript for publication, providing that you modify the manuscript according to the review recommendations.

As you will see, the revised version of this manuscript has been examined by 3 reviewers. The reviewers came to widely differing views, including a recommendation to reject the submission. After extensive consideration, the editorial view is that you should be given an opportunity to respond to these comments in a further round of revision. Whilst minor points of revision are raised by Reviewer #3, Reviewer #1 points out that in the absence of any data showing that DOC2.1 binds PS, there is now no obvious link between the DOC2.1 data in the revised manuscript and the data on the ATP2B/CDC50.4 complex. We agree that this substantially reduces the impact and breadth of the manuscript since it means that ther is now no experimentally validated functional link between DOC2.1 function and the function of the ATP2B/CDC50.4 complex. This conclusion is further strengthened by the fact that mutagenesis of DOC2.1 residues presumed to be involved in calcium binding affected egress but not invasion or microneme discharge. We would be grateful if you could address this major comment, as well as those of Reviewer #3. In addition, it is noted that the localisation by immunofluorescence of CDC50.4-mAID-HA in panels E and F of Figure 2 appears different from that of HA-tagged CDC50.4 (Fig 2B). Can you please address this issue too?

Reviewer Comments (if any, and for reference):

Reviewer's Responses to Questions

**Part I - Summary**
Please use this section to discuss strengths/weaknesses of study, novelty/significance, general execution and scholarship.

Reviewer #1: The authors have dealt with the reviewer comments comprehensively. It is good that they decided to pull back on the direct interaction of DOC2.1 and PS in light of further work being needed to substantiate this.

We thank the reviewer for the expertise and suggestions.

Reviewer #2: The revised manuscript by Bisio and colleagues incorporates several of the requested experiments and changes. However, some major comments were not addressed by the authors due to technical difficulties, even for experiments previously performed and published by the lab, such as assessing the involvement of the ATPases identified in flipping phospholipids other than PS.

As mentioned in the previous revision, the assessment of other phospholipids like PA or PC is challenging due to the low intrinsic flipping activity in *Toxoplasma gondii*. As indicated by the reviewer, we have previously generated and published such results as shown below (Bisio, 2019). The graph represents the increase in non-quenchable lipids upon time (as described in the manuscript):

As depicted in the figure included as supplementary material in (Bisio, 2019), levels of internalization of PA and PC are (at least) 6 time lower than PS, and limits the resolution needed in order to identify proteins involved in such processes. While it is still possible to performed such experiments for these phospholipids, we have opted to focus on PS, since robust flipping activity can be obtained.

Bisio H, Lunghi M, Brochet M, Soldati-Favre D. Phosphatidic acid governs natural egress in Toxoplasma gondii via a guanylate cyclase receptor platform. Nat Microbiol. 2019;4(3):420-8.

They also have not been able to reproduce consistently the DOC2.1 liposome binding experiments. As a result, the authors have removed a significant portion of the results section relating to DOC2.1. This is a very honest response, which I highly value, however, this leave the manuscript in a weaker position and slightly disjointed.

We are aware that the limitation on the experimental setup has weakened the manuscript and the link between DOC2.1 and PS is now merely hypothetical. As stated in the previous revision, we have made a considerable effort to produce and purify DOC2.1 recombinantly, but it has resulted in insoluble protein. We have now further revisited the manuscript and all data related to DOC2.1 has been removed from the manuscript in order to simplify the message. In the current version of the manuscript, only at the discussion section, we have briefly hypothesized about a possible role of C2-containing proteins in the binding of PS.

As the manuscript currently stands, the authors have convincingly shown that ATP2A and ATP2B bind to CDC50.4, and that ATP2B and CDC50.4 play an important role in egress, invasion and microneme secretion. They also show that knockdown of these two proteins leads to a block in PS flipping. While it is possible that ATP2B is directly involved in PS flipping, this has not been shown experimentally.

While we agree with the reviewer, the assessment of direct flipping by ATP2B would require protein production and biochemical analysis of the recombinant protein, which is rather challenging for polytopic membrane proteins. Moreover, we would like to highlight that the ATP2 from *P. chabaudi*, homologue of *T. gondii* ATP2B and ATP2A, has been experimentally demonstrated to flip phosphatidylserine and phosphatidylethanolamine using purified recombinantly-produced protein (Lamy, 2021). Consequently, taken together with the data provided in this manuscript, it is parsimoniously sound to hypothesize that the block in PS flipping upon depletion of ATP2B is a direct effect.

Lamy A, Macarini-Bruzaferro E, Dieudonne T, Peralvarez-Marin A, Lenoir G, Montigny C, et al. ATP2, The essential P4-ATPase of malaria parasites, catalyzes lipid-stimulated ATP hydrolysis in complex with a Cdc50 beta-subunit. Emerg Microbes Infect. 2021;10(1):132-47.

The authors also show that ATP7B is important for lytic growth, but do not characterise this line further.

As stated in the previous revision, ATP7B is not the main focus of this manuscript and the phenotypic characterization presented reflects such statement. Importantly, the lack of plasma membrane localization of ATP7B impedes the identification of the phospholipid(s) flipped by such protein using the methods described in this manuscript. Moreover, the phenotype associated with intracellular growth (as shown here) and extracellular survival (Primo, 2021), are not linked to microneme exocytosis, which is the main focus of the manuscript. Overall, we believe that a further characterization of ATP7B falls out of the scope of the manuscript.

Primo VA, Rezvani Y, Farrell A, Vajdi A, Marth GT, Zarringhalam K, et al. The extracellular milieu of *Toxoplasma*’s lytic cycle drives lab-adaptation and promotes changes in lipid metabolism primarily driven by transcriptional reprogramming. bioRxiv. 2021:2021.03.09.434570.

Finally, the authors show that knockdown of DOC2.1, a protein thought to be involved in microneme secretion, leads to a block in lytic growth due to the inability of the parasites to egress, invade or secrete micronemes. By mutating the calcium binding sites of DOC2.1, they also show that these sites seem to be important for egress but not invasion or microneme secretion. This is a surprising result, but not further pursued.

We agree with the reviewer that these results are surprising and a rather interesting venue for future research. Importantly, the study of mechanistic details associated with such differential function of DOC2.1 would require a significant amount of work, likely including identification of feedback loops, slight changes in the activity of proteins involved in microneme exocytosis upon extracellular or intracellular conditions and/or the identification of interacting partners for DOC2.1 and their temporal interaction. We believe that such in-depth studies of the pleiotropic functions of DOC2.1 and the signaling pathway associated with it, fall out of the scope of the manuscript. Concordantly, we have removed all data related to DOC2.1.

Since the authors removed the DOC2.1 liposome binding experiments, which showed binding of DOC2.1 to PS, there is no longer a link between the role of DOC2.1 and ATP2B/CDC50.4. This weakens the manuscript significantly.

As previously stated and in order to avoid confusion of the reader, we have opted to remove all data related to DOC2.1 from the manuscript.

Another concern is that in Figure 2, the localisation of CDC50.4-HA in panel B appear to occupy a very different localisation compared to CDC50.4-mAID-HA in panel E. This seems to indicate that mAID-tagging of CDC50.4 mislocalises the protein.

We thank the reviewer for noticing this inconsistency in the localization of CDC50.4-mAID-HA. Importantly, all immunofluorescence shown in this manuscript for CDC50.4 corresponds to the -mAID-HA tagged version of the protein. In consequence, the changes observed in the localization of the protein are not due to mistargeting of the protein upon tagging with mAID but rather the combination of the tagging of ATP2A and CDC50.4.

We have now repeated the transfection to introduce a 3xTy tag in the genomic locus of ATP2A in CDC50.4-mAID-HA strain, in order to assess localization of CDC50.4 prior or after ATP2A endogenous tagging. Importantly, while CDC50.4 possesses its reported apical localization in the parental CDC50.4-mAID-HA line, tagging of ATP2A renders the protein with a plasma membrane localization, no longer localized at apical tip (Figure 2E). Expression of full-length proteins was assessed by western blot (Figure S2F). We have now commented on such results in the manuscript as follows:

‘Importantly, double tagging of ATP2A and CDC50.4 rendered a partial miss localization of the complex (Figure 2E), indicating some steric impediment for correct trafficking upon presence of both C-terminal tags.’

Reviewer #3: This work studies the role of a complex formed between the flippase ATP2B with the cell division control protein (CDC) 50.4 for the flipping and uneven distribution of phosphatidylserine, which may act as lipid mediator for organelle fusion and microneme exocytosis. Authors demonstrated the interaction of CDC50.4 with ATP4B and focused the work on the function of these proteins. Downregulation of CDC50.4 led to degradation of ATP2B and the complex was critical for T. gondii growth. Invasion and egress was impaired in the mutants while intracellular replication was not. Microneme secretion of extracellular parasites was reduced upon downregulation of either ATP2B or CDC50.4. The authors claim that DOC2.1, a previously described key egress and invasion factor, senses changes in cytosolic calcium in intracellular parasites and may be the plausible sensor of PS at the inner leaflet of the plasma membrane.
This work is interesting, and it is an important contribution to our knowledge of the role of P4-ATPases flippases in T. gondii. Authors responded to most of the previous critique of this reviewer. Two minor concerns are indicated.

We thank the reviewer for the expertise and suggestions.

**Part II – Major Issues: Key Experiments Required for Acceptance**
Please use this section to detail the key new experiments or modifications of existing experiments that should be absolutely required to validate study conclusions.

Generally, there should be no more than 3 such required experiments or major modifications for a "Major Revision" recommendation. If more than 3 experiments are necessary to validate the study conclusions, then you are encouraged to recommend "Reject".

Reviewer #1: (No Response)

Reviewer #2: (No Response)

Reviewer #3: No major issues

**Part III – Minor Issues: Editorial and Data Presentation Modifications**
Please use this section for editorial suggestions as well as relatively minor modifications of existing data that would enhance clarity.

Reviewer #1: (No Response)

Reviewer #2: (No Response)

Reviewer #3: Two minor concerns that could easily be clarified in the legends to the figures.
The legend for Figure 2C needs an explanation for how the westerns were done. It is mentioned the IPs with anti-Ty but the antibodies used for the western are not mentioned.

We apologize for the lack of information. The figure legend has been updated. We have also noticed that a section describing the IP in materials and methods was lacking, and it has now been included. We thank the reviewer for the comments.

The PCRs presented in FigS4B are a little confusing. Could the authors include the size of the expected bands? There is a shadow/smear in part B. Does it mean anything? Please clarify what is expected and the result.
This figure is important to understand how the mutation of DOC2.1 was made.

We apologize for the lack of information. Regardless, all data related to DOC2.1 has now been removed from the article.

PLOS authors have the option to publish the peer review history of their article ([what does this mean?](https://eu-central-1.protection.sophos.com?d=outlook.com&u=aHR0cHM6Ly9ldXIwMy5zYWZlbGlua3MucHJvdGVjdGlvbi5vdXRsb29rLmNvbS8_dXJsPWh0dHBzJTNBJTJGJTJGam91cm5hbHMucGxvcy5vcmclMkZwbG9zcGF0aG9nZW5zJTJGcyUyRmVkaXRvcmlhbC1hbmQtcGVlci1yZXZpZXctcHJvY2VzcyUyM2xvYy1wZWVyLXJldmlldy1oaXN0b3J5JmRhdGE9MDQlN0MwMSU3QyU3QzcwNzAwZDFjZGRkZDQyYWY0ZjM5MDhkOWNhOWNhMDFlJTdDNGVlZDc4MDdlYmFkNDE1YWE3YTk5MTcwOTQ3ZjRlYWUlN0MwJTdDMCU3QzYzNzc2MzU5NjMzOTk4ODQ1NSU3Q1Vua25vd24lN0NUV0ZwYkdac2IzZDhleUpXSWpvaU1DNHdMakF3TURBaUxDSlFJam9pVjJsdU16SWlMQ0pCVGlJNklrMWhhV3dpTENKWFZDSTZNbjAlM0QlN0MzMDAwJnNkYXRhPVglMkZwbDQzN1BxTFZFYzlYdGFDWFhXbFNKbEQ2V09WekxSRllMVlN6ZXdOMCUzRCZyZXNlcnZlZD0w&i=NjEyM2Q2NzY3Yjc5YTIwZjM5MzZhZjY1&t=VWdCRXlZTnNkVFFQZzlsbGhYaUlxL3p1SzFhdlJYYXZPektlbHltQ2I2OD0=&h=bb10f6cb279b4aeb94213f838f542038)). If published, this will include your full peer review and any attached files.


If you choose “no”, your identity will remain anonymous but your review may still be made public.


**Do you want your identity to be public for this peer review?** For information about this choice, including consent withdrawal, please see our [Privacy Policy](https://eu-central-1.protection.sophos.com?d=outlook.com&u=aHR0cHM6Ly9ldXIwMy5zYWZlbGlua3MucHJvdGVjdGlvbi5vdXRsb29rLmNvbS8_dXJsPWh0dHBzJTNBJTJGJTJGd3d3LnBsb3Mub3JnJTJGcHJpdmFjeS1wb2xpY3kmZGF0YT0wNCU3QzAxJTdDJTdDNzA3MDBkMWNkZGRkNDJhZjRmMzkwOGQ5Y2E5Y2EwMWUlN0M0ZWVkNzgwN2ViYWQ0MTVhYTdhOTkxNzA5NDdmNGVhZSU3QzAlN0MwJTdDNjM3NzYzNTk2MzM5OTg4NDU1JTdDVW5rbm93biU3Q1RXRnBiR1pzYjNkOGV5SldJam9pTUM0d0xqQXdNREFpTENKUUlqb2lWMmx1TXpJaUxDSkJUaUk2SWsxaGFXd2lMQ0pYVkNJNk1uMCUzRCU3QzMwMDAmc2RhdGE9bE8lMkZGV3o2dnRUd05XZzk5dnVPMGRoTzlSaElBbTFZNXRjRlB1WTJmSWFFJTNEJnJlc2VydmVkPTA=&i=NjEyM2Q2NzY3Yjc5YTIwZjM5MzZhZjY1&t=TStOaGJQZUQwc0ZPU3B1MmpFYlpaOWdmWWNWdllsTzQ0UG9XeUlKc3lRMD0=&h=bb10f6cb279b4aeb94213f838f542038).

Reviewer #1: No

Reviewer #2: No

Reviewer #3: No
